# Aggressive Burkitt Lymphoma Mimicking Acute Pancreatitis: A Case Report

**DOI:** 10.3390/reports9020103

**Published:** 2026-03-27

**Authors:** Nicole Sequeira, Rachael Hagen, Chidambaram Ramasamy, Poolakkad S. Satheeshkumar, Kapil Meleveedu

**Affiliations:** 1 Department of Medicine, University of Connecticut Health Center, Farmington, CT 06030, USA; 2 Division of Hematology and Oncology, Department of Medicine, University of Connecticut Health Center, Farmington, CT 06030, USA; 3 Division of Hematology and Oncology, Department of Medicine, Jacobs School of Medicine and Biomedical Sciences, University of Buffalo, Buffalo, NY 14203, USA

**Keywords:** Burkitt lymphoma, case report, non-Hodgkin lymphoma, pancreatitis, retroperitoneal mass, testicular lymphoma

## Abstract

**Background and Clinical Significance**: Burkitt lymphoma is an aggressive form of non-Hodgkin lymphoma of B-cell origin, caused by a *MYC* gene translocation on chromosome 8. There are three clinical subtypes, of which the sporadic subtype is most prevalent in the United States. Sporadic Burkitt lymphoma is diagnosed at a median age of 30 years and commonly manifests as bulky abdominal lesions, most often involving the ileocecal region. Pancreatic involvement is uncommon, and presentation as acute pancreatitis secondary to Burkitt lymphoma is exceedingly rare. **Case Presentation**: We present a case of a young male who presented with epigastric pain, nausea, and vomiting. He had a diffusely tender abdomen and elevated lipase levels. On imaging, he was found to have large retroperitoneal and intraperitoneal masses, contiguous with an enlarged pancreas. Burkitt lymphoma was confirmed upon biopsy of duodenal and gastric masses via endoscopic ultrasound. MRI brain and testicular ultrasound revealed unilateral fifth cranial nerve and bilateral testicular involvement, respectively. His course was complicated by bowel perforation requiring urgent surgery. However, he achieved complete remission with dose-dense systemic and intrathecal chemotherapy. **Conclusions**: This case highlights the diverse presentations of Burkitt’s lymphoma and a favorable prognosis with treatment. Clinicians should maintain a high index of suspicion for a malignant etiology of acute pancreatitis in patients without classic risk factors.

## 1. Introduction and Clinical Significance

Burkitt lymphoma (BL) is a rare and aggressive type of non-Hodgkin lymphoma. It is among the most rapidly growing human tumors with a doubling time of 24–48 h. In 2001, the World Health Organization (WHO) classified BL into three clinical subtypes: endemic, sporadic, and immunodeficiency-related [1]. Sporadic BL is the most frequently observed subtype in the United States, with an incidence of approximately three cases per million people per year [2]. Unlike the endemic form, which is strongly associated with the Epstein–Barr virus (EBV), EBV positivity is seen in only 20–30% of cases of the sporadic subtype.

Clinically, 60–80% of patients present with bulky abdominal lesions, often involving the terminal ileum. Bone marrow involvement occurs in approximately 30% of cases, while central nervous system (CNS) infiltration is observed in around 15% [3]. Pancreatic involvement is uncommon. When present, BL typically occurs in the pancreatic head, leading to obstructive jaundice [4]. Acute pancreatitis as an initial presentation of BL is rare, described only in a limited number of case reports, with a greater prevalence in pediatric populations [5]. Here, we describe a case of a young adult who developed acute pancreatitis as the first manifestation of sporadic BL.

## 2. Case Presentation

A 23-year-old Hispanic male with a history of lumbar disk herniations from a motor vehicle accident two years prior presented to the emergency room with one week of nausea, vomiting, and abdominal pain. He initially started having nausea after meals, which progressed to severe abdominal cramping and vomiting. The pain was primarily epigastric, radiated to his chest, and worsened with food intake. He also reported a 10-pound weight loss over 10 days, right facial numbness, and right flank pain. Although he had back pain, it felt consistent with the chronic lower back pain from his motor vehicle accident, without a significant change from baseline. On further questioning, he reported a six-month history of night sweats. He had no significant family history and denied a family history of cancer. He reported daily marijuana use and occasional alcohol use but denied nicotine use. Physical exam revealed diffuse abdominal tenderness, particularly in the right lower quadrant. Laboratory findings were notable for leukocytosis (13.3 × 10^3^/μL, normal range: 3.8–10.6 × 10^3^/μL) with left shift, elevated lipase (402 U/L, normal range: 8–51 U/L), and elevated lactate dehydrogenase (LDH) (1733 U/L, normal range: 125–230 U/L), consistent with acute pancreatitis.

**Figure 1:**
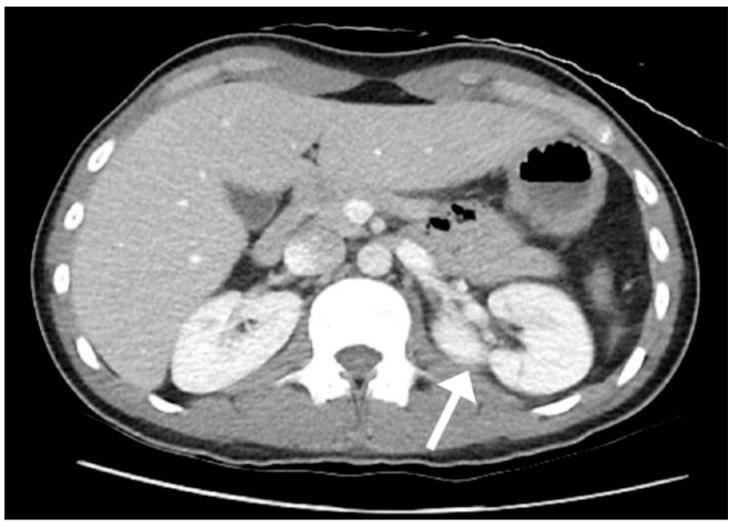
CT of the abdomen and pelvis revealed large infiltrating retroperitoneal and intraperitoneal masses encasing the left kidney (white arrow), contiguous with the enlarged pancreas, with moderate fluid. A pancreatic head mass and dilated pancreatic duct were also seen.

Although he satisfied the diagnostic criteria for acute pancreatitis, the absence of typical risk factors of acute pancreatitis, along with the presence of right lower quadrant tenderness and an elevated LDH, suggested an atypical presentation and prompted further imaging. Computed tomography (CT) of the abdomen and pelvis showed a large retroperitoneal mass encasing the pancreas, left kidney, and renal vasculature, a mass in the right lower abdominal cavity, diffuse thickening of the stomach wall, and moderate complex free fluid (Figure 1). The pancreatic duct was dilated up to 4 mm with an abrupt cutoff at the level of a 2.5 cm pancreatic head mass. His presentation and imaging were concerning for a malignant process. At this point, the differentials considered were primary pancreatic malignancies such as pancreatic adenocarcinoma, primary pancreatic lymphoma, and aggressive B-cell lymphomas such as Burkitt lymphoma. Metastatic malignancy with unknown primary, germ cell tumors and sarcoma were other possibilities. Given the patient’s dramatic symptomatology, massive abdominal involvement, and elevated LDH, lymphoma was considered most likely.

**Figure 2:**
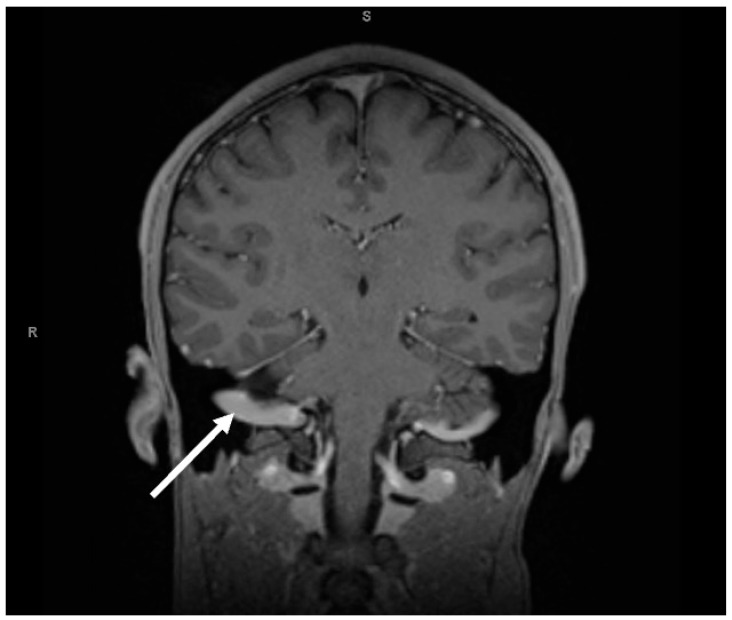
MRI brain showed a mass arising from the right fifth cranial nerve (white arrow), involving the right ganglion and maxillary divisions, as well as the cisternal portion of the right cranial nerve five.

The patient was admitted to the hospital for further workup, staging and treatment. CT of the chest was negative for suspicious pulmonary masses as well as mediastinal, hilar, and axillary lymphadenopathy. CT of the head was negative for an acute intracranial process. On the second day of the hospital admission, given persistent right-side facial numbness and a negative head CT, Magnetic Resonance Imaging (MRI) of the brain was done, which showed a mass arising from the right fifth cranial nerve (Figure 2).

**Figure 3:**
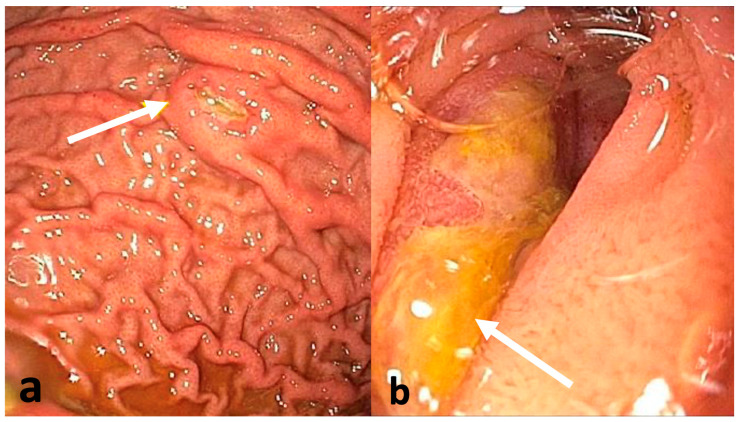
(**a**) EGD revealed ulcerated gastric tumors (white arrow) on the greater and lesser curvatures of the stomach and (**b**) an ulcerated mass (white arrow) in the third portion of the duodenum.

**Figure 4:**
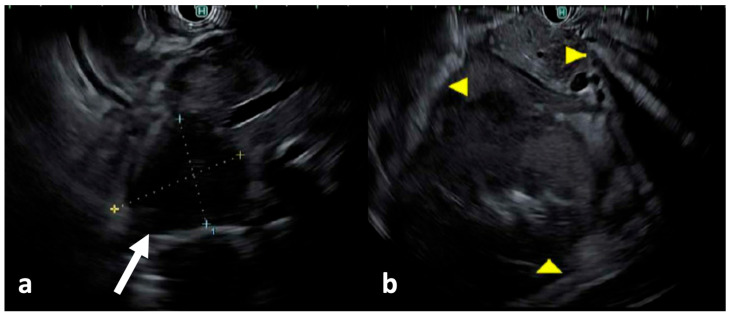
(**a**) EUS showed a 2.5 cm × 3.0 cm pancreatic head mass (white arrow, dotted lines), and (**b**) enlarged peripancreatic lymph nodes (yellow arrows) encasing the left kidney, measuring up to 3.5 cm × 3.8 cm.

The same day, the patient underwent esophagogastroduodenoscopy (EGD), which demonstrated medium-sized non-circumferential ulcerated masses on the greater and lesser curvatures of the stomach (Figure 3a), and a medium-sized infiltrative submucosal ulcerated mass in the third portion of the duodenum (Figure 3b). Biopsies were taken with cold forceps. Endoscopic ultrasound (EUS) revealed a 2.5 cm × 3.0 cm pancreatic head mass (Figure 4a) and peripancreatic lymphadenopathy (Figure 4b). Fine needle aspiration for cytology and fine needle biopsy of the enlarged peripancreatic lymph nodes were performed. After the preliminary pathology report indicated lymphoma, he underwent a testicular ultrasound to evaluate for a potential sanctuary site, which showed hypoechoic foci in the bilateral testes, suggestive of testicular involvement. These investigations were completed in the order described within the first four days of hospital admission. The patient was at high risk of tumor lysis syndrome (TLS) due to the aggressive nature of the malignancy and rapid doubling time. The preventative measures taken against TLS included twice daily labs, intravenous fluids, and allopurinol 300 mg twice daily.

The endoscopic biopsies were evaluated with Hematoxylin and Eosin (H&E) staining, immunohistochemistry (IHC), and Fluorescent In Situ Hybridization (FISH). IHC demonstrated a CD10+, CD19+, CD20+ high-grade aggressive B-cell lymphoma with approximately 100% Ki-67 proliferation index. The morphologic and immunophenotypic findings were consistent with a high-grade, aggressive CD10+ B-cell lymphoma.

**Figure 5:**
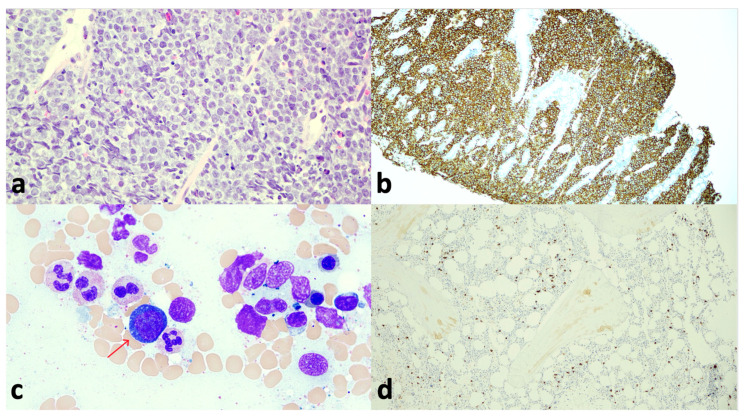
(**a**) Duodenal biopsy (H&E, 40×) showed lamina propria infiltration by a population of monomorphic, medium-sized lymphoid cells with round to irregular nuclei with finely clumped and dispersed chromatin, and multiple variably prominent nucleoli. Mitoses and apoptosis are noted. (**b**) Duodenal biopsy revealed neoplastic cells staining positive for CD20 by IHC (40×). The gastric and retroperitoneal lymph node biopsies demonstrated the same characteristics. (**c**) Bone marrow aspirate (Wright–Giemsa stain, 60×) showed normocellular bone marrow with rare atypical lymphoid cells (red arrow). (**d**) Bone marrow biopsy demonstrated a few single, scattered CD20-positive B-cells on IHC.

Interestingly, the neoplastic cells showed weak to strong expression of BCL-2 on IHC, further complicating the diagnostic picture. Per the 2008 WHO classification, weak BCL-2 expression is seen in about 20% cases and does not exclude the diagnosis, but strong expression of BCL-2 is not compatible with a diagnosis of BL. The differential diagnosis at that point included diffuse large B-cell lymphoma or high-grade B-cell lymphoma with *MYC* and *BCL-2* rearrangements, high-grade B-cell lymphoma (not otherwise specified), and high-grade B-cell lymphoma with 11q aberration. FISH later revealed *MYC* positivity with no evidence of *BCL-2* or *BCL-6* rearrangements. This profile was most consistent with Burkitt lymphoma, despite atypical BCL-2 expression on IHC (Figure 5a,b).

The patient had a normocellular bone marrow on biopsy (Figure 5c,d) with a minute subset of CD10+ B-cells (<0.1% of total cells) showing lambda light chain restriction on flow cytometry, indicative of very low-level involvement by B-cell lymphoma. According to the 2025 National Comprehensive Cancer Network (NCCN) guidelines, morphologic evidence of any lymphoma cells in the bone marrow aspirate is sufficient for a diagnosis of Stage IV disease. Human Immunodeficiency Virus (HIV), hepatitis B, and hepatitis C serologies were negative. He was positive for the IgG antibody to the EBV nuclear antigen, indicating previous exposure, but not to the viral capsid antigen or early D antigen. He was ultimately diagnosed with stage IV Burkitt lymphoma, with confirmed involvement of the CNS, pancreas, gastrointestinal tract, bone marrow, and testes.

By the fifth day of the hospital admission, Burkitt lymphoma was considered likely based on preliminary pathology results (IHC had resulted by this time, but the flow cytometry and bone marrow results were pending). The Oncology team had planned to initiate chemotherapy the next day. Unfortunately, on the morning he was supposed to start chemotherapy, he developed an acute abdomen with imaging suggestive of a perforation. He was taken to the OR for an exploratory laparotomy, where he was found to have a perforation of his cecum with a tumor involving the right colon. He underwent a right hemicolectomy with end ileostomy. He was unable to start chemotherapy on the day of the surgery due to his critical condition. The next day, he was urgently initiated on chemotherapy with R-CODOX-M/R-IVAC, while still awaiting FISH studies for *MYC* and *BCL-2*. This regimen is a modified version of the original Magrath regimen, consisting of alternating cycles of CODOX-M and IVAC, for a total of four cycles. R-CODOX-M consisted of rituximab 375 mg/m^2^ on day 0, cyclophosphamide 800 mg/m^2^ on days 1 and 2, vincristine 1.5 mg/m^2^ on days 1 and 10, doxorubicin 50 mg/m^2^ on day 1, and methotrexate 3000 mg/m^2^ on day 10. R-IVAC consisted of rituximab 375 mg/m^2^ on day 0, ifosfamide 1500 mg/m^2^ on days 1–5, etoposide 60 mg/m^2^ on days 1–5, and cytarabine 2000 mg/m^2^ on days 1 and 2. An Ommaya reservoir was inserted, and he was started on intrathecal chemotherapy, receiving a total of four infusions of methotrexate (12 mg) and nine infusions of cytarabine (50 mg).

**Figure 6:**
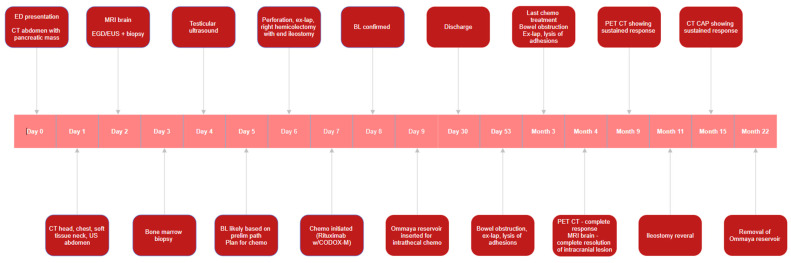
Timeline of events from presentation to two-year follow-up.

His cerebrospinal fluid (CSF) was positive for B-cell lymphoma on flow cytometry but cleared after six days of treatment. His hospital admission was further complicated by surgical site infection with superficial wound dehiscence and febrile neutropenia, both of which resolved with antibiotics and supportive care. He was eventually discharged from the hospital 30 days after admission. Four weeks later, he developed small bowel obstruction (SBO), requiring urgent laparotomy with lysis of adhesions, with a second episode of SBO two months after. Despite these challenges, he responded well to chemotherapy and completed treatment three months after presentation. Whole-body Positron Emission Tomography (PET) scan one month after treatment completion revealed a complete metabolic response. MRI brain done at the same time demonstrated resolution of the intracranial enhancing lesion. Surveillance consisted of CT of the chest, abdomen and pelvis performed at six-month intervals for one year, in accordance with the NCCN guidelines. Surveillance scans showed no evidence of recurrent disease. He underwent ileostomy reversal eleven months after it was created. The patient remains cured two years later with a complete return to baseline functional status. The chronologic sequence of events is summarized in Figure 6.

## 3. Discussion

Sporadic BL commonly involves the lymph nodes and abdomen, with a predilection for the lymphatic-rich ileocecal area. Pancreatic involvement is rare, with most reported cases occurring in pediatric populations. This case highlights a classic presentation of acute pancreatitis in a young male without traditional risk factors. Imaging revealed an enlarged pancreas contiguous with a retroperitoneal mass. In cases of adults with BL presenting as acute pancreatitis, approximately half showed distinct pancreatic masses on imaging. The remaining cases involved either diffuse pancreatic enlargement or tumors indistinguishable from the pancreas. Obstructive jaundice was present in about half of the patients [4,5,6,7,8,9,10,11,12].

Cases of BL with pancreatic involvement in adults reported in the literature are summarized in Table 1. Case reports were sourced from PubMed using search terms “Burkitt lymphoma” and “pancreas,” “pancreatic involvement,” or “pancreatitis,” covering publications through July 2025. Each retrieved article was then individually examined to identify cases involving adult patients with primary pancreatic disease. The present case is unique in the widespread involvement (pancreas, CNS, GI tract, bone marrow, and testes), and favorable outcome with systemic and intrathecal chemotherapy at the end of a two-year follow-up period.

Diagnosis of acute pancreatitis requires at least two of three criteria: characteristic abdominal pain, lipase or amylase ≥ 3 times the upper limit of normal, and imaging suggestive of pancreatitis. In this case, the patient had the typical epigastric abdominal pain and lipase elevation sufficient to diagnose pancreatitis. However, he had a variety of clinical findings that were not consistent with classic presentations of pancreatitis—namely, facial numbness, right lower quadrant pain, and elevated LDH. Imaging was essential to identify the underlying malignancy. Notably, non-specific diffuse pancreatic enlargement on imaging is consistent with pancreatitis and can mask the diagnosis of BL, delaying treatment.

Contrast-enhanced CT is the imaging modality of choice for acute pancreatitis [13]. In BL, CT often shows peritoneal thickening, discrete or conglomerated masses, widespread lymphadenopathy, and abdominal tumors. There is typically infiltrative growth with involvement of retroperitoneal lymph nodes and encasement of mesenteric vessels. These findings were similarly seen in our patient’s case, with diffuse lymphadenopathy and contiguous tumors invading the pancreas, kidney, and vasculature. It is also important to evaluate for testicular or ovarian involvement given the predilection for extranodal involvement, as seen in this patient.

Despite its aggressive nature, the prognosis of BL is favorable when treated with chemotherapy, emphasizing the importance of early recognition to expedite treatment. Given its rapid doubling time, BL requires intensive therapy over a short period of time. Adult treatment regimens are extrapolated from pediatric trials but report inferior outcomes [14]. Nevertheless, with dose-dense therapy, 65–100% of patients achieve a complete response, with overall survival rates of 50–70% [15]. However, CNS involvement is associated with a poor prognosis, highlighting the need for continued research to improve outcomes [16].

When evaluating patients with acute pancreatitis without a clear etiology, the differential diagnosis should be broadened and revisited to prompt further workup. A thorough history is critical, such as inquiring about constitutional symptoms and involvement of other organs, particularly abdominal organs and the testicles or ovaries. Contrast-enhanced abdominal CT should be considered and may reveal findings suggestive of BL. LDH is a less invasive and cheaper laboratory test that can also raise suspicion for an oncological process if elevated. This case emphasizes the importance of maintaining a broad differential and a high index of suspicion in patients without classic features of pancreatitis, especially given the favorable prognosis with chemotherapy, underscoring the importance of early recognition to expedite treatment.

## 4. Conclusions

In conclusion, BL should be suspected in patients with bulky abdominal and lymph node masses, elevated LDH, and, rarely, pancreatitis from pancreatic involvement. This case was unique in the involvement of multiple organ systems at presentation, including the pancreas, fifth cranial nerve, and bilateral testes. It also underscores the importance of imaging to determine the etiology of pancreatitis in a young patient with no obvious risk factors. As presentation can be severe with rapid onset, suspected BL necessitates an expedited workup under the guidance of oncology and should be considered a medical emergency [16]. Prompt diagnosis with cross-sectional imaging and biopsy is crucial for a favorable outcome, given the rapid proliferation of this aggressive yet curable cancer.

## 5. Patient Perspective

The patient provided his perspective below;

*“The symptoms felt like a combination of illnesses; exhaustion that couldn’t be satisfied with sleep, sporadic numbness, loss of appetite, constant nausea and vomiting, abdominal pain, and weight loss. Of course, with the chemotherapy, I lost even more weight and grew even more exhausted, but the overall pain slowly left. Common side effects (nausea) were also present. After treatment, aside from related complications, I had a swift recovery. Two months after my final discharge, I was able to return to work. A total of six months completely reversed all symptoms I had prior to treatment. Two years after everything happened, I’m as close to 100% as I can be. I’m back to exercising just the same and working just as hard.”*

## Figures and Tables

**Table 1:** Burkitt lymphoma with pancreatic involvement in adult patients reported in the literature.

	Year	Author	Age/Sex	HIV/EBV	Lipase (U/L)	Imaging Findings	IHC	CNS	Treatment	Outcome
1	1982	Francis [4]	59/F	U/U	High	Enlarged pancreas	U	NI	U	U
2	2001	Sainz [5]	32/M	+/U	184	Retroperitoneal masses surrounding the peripancreatic tissue	U	NI	U	U
3	2005	Novoselac [6]	84/M	U/U	U	Pancreatic head mass, biliary ductal dilation	CD20+, C10+, CD5-	NI	U	U
4	2008	Chang [7]	34/F	+/U	1172	Interstitial pancreatitis	t (8; 14) (q24; q32)	NI	U	U
5	2012	Bacchi [8]	76/M	-/-	136	Enlarged pancreas with infiltrative tissue	CD20+, C10+; Ki-67 100%. CD2-	NI	None	Died 1 h after biopsy
6	2013	Nakaji [9]	86/M	U/-	U	Pancreatic head mass	CD20+, CD10+; Ki-67 ~100%. t (8; 14) (q24; q32)	NI	U	U
7	2014	Carbonetti [10]	64/F	-/-	6872	Enlarged pancreatic head and peripancreatic lymph nodes	CD20+, C10+; Ki-67 > 90%. t (8; 14)	NI	Prednisolone, vincristine, daunorubicin, L-asparaginase, methotrexate	Died at 7 months
8	2017	Rwegerera [11]	22/F	+/+	U	Enlarged pancreas and peri-pancreatic lymph nodes, pancreatic head mass	CD10+, CD20+; Ki-67 100%. Bcl-6-, Bcl-2-, CD3-, MUM1-	NI	None	Died day 12 of admission
9	2018	Konjeti [12]	68/F	U/U	U	Periportal hepatic vs. pancreatic mass	CD20+, C10+. Bcl-6+. Ki-67 > 95%.	I	Etoposide, prednisone, vincristine, doxorubicin	Died after 2 cycles of chemotherapy
10	2026	Present case	23/M	-/-	402	Retroperitoneal mass encasing the pancreas, pancreatic head mass	CD10+, CD19+, CD20+. Ki-67 ~100%. c-Myc rearrangement. Bcl-2-, Bcl-6- by FISH.	I	Rituximab, cyclophosphamide, doxorubicin, vincristine, methotrexate, iphosphamide, etoposide, cytarabine	Complete response/Alive at 2-year follow-up

**Abbreviations**: I: involvement; IHC: immunohistochemistry; NI: no involvement; U: unknown.

## Data Availability

The original contributions presented in this study are included in the article. Further inquiries can be directed to the corresponding author.

## Abbreviations

BL	Burkitt lymphoma
CNS	Central nervous system
CT	Computed Tomography
EBV	Epstein–Barr virus
EGD	Esophagogastroduodenoscopy
EUS	Endoscopy ultrasonography
HIV	Human Immunodeficiency Virus
LDH	Lactate dehydrogenase
MRI	Magnetic Resonance Imaging
PET	Positron Emission Tomography
WHO	World Health Organization
